# Silencing of Sly-miR171d increased the expression of *GRAS24* and enhanced postharvest chilling tolerance of tomato fruit

**DOI:** 10.3389/fpls.2022.1006940

**Published:** 2022-09-09

**Authors:** Zengting Xing, Taishan Huang, Keyan Zhao, Lanhuan Meng, Hongmiao Song, Zhengke Zhang, Xiangbin Xu, Songbai Liu

**Affiliations:** ^1^School of Food Science and Engineering, Hainan University, Haikou, China; ^2^Suzhou Key Laboratory of Medical Biotechnology, Suzhou Vocational Health College, Suzhou, China

**Keywords:** Sly-miR171d, tomato, chilling tolerance, GRAS, GA

## Abstract

The role of Sly-miR171d on tomato fruit chilling injury (CI) was investigated. The results showed that silencing the endogenous Sly-miR171d effectively delayed the increase of CI and electrolyte leakage (EL) in tomato fruit, and maintained fruit firmness and quality. After low temperature storage, the expression of target gene *GRAS24* increased in STTM-miR171d tomato fruit, the level of GA_3_ anabolism and the expression of *CBF1*, an important regulator of cold resistance, both increased in STTM-miR171d tomato fruit, indicated that silencing the Sly-miR171d can improve the resistance ability of postharvest tomato fruit to chilling tolerance.

## Introduction

Low temperature storage technology can reduce respiratory metabolism and is widely used in prolonging fruit storage period (Duan et al., 2022). However, fruit from subtropical and tropical regions stored below the temperature of 12°C are susceptible to the risk of chilling injury (CI), resulting in lower product quality and lower sales (Ding et al., 2021). Tomato fruit is the major contributors of minerals, dietary fiber, sugars, essential amino acids, and vitamins in the daily diet (Bai et al., 2020). It widely distributes in tropical and subtropical regions. However, tomato fruit stored at low temperature below 10°C (green ripening stage) and 5°C (red ripening stage) for a long time are susceptible to CI. During storage at low temperatures, incipient CI in tomato is not generally apparent. After returning the fruit to room temperature, CI is very obvious, such as failure to ripen properly, indentation of the surface or damage, discoloration, decay and increased water loss (Tian et al., 2022). CI sharply led to a decline in tomato quality and consumer acceptance, which ultimately led to huge economic losses (Luengwilai et al., 2012). Hence, controlling the postharvest CI of fruit is essential to ensure good quality and minimize losses.

MicroRNAs (miRNAs) is a class of endogenous non-coding small RNAs with a length of about 18–25 nt (Bartel, 2004). The miRNAs binds to mRNA molecules through base pairing, resulting in translation inhibition or cleavage of its target mRNA (Barciszewska-Pacak et al., 2015). Negative regulation of target genes by miRNAs is critical for coordinating the plant responses to cold stress (Zhao et al., 2022b). In recent years, miRNAs has attracted more and more attention in their important role in the post-transcriptional or translational regulation of gene expression and plant stress tolerance (Sunkar et al., 2007). In rice (*Oryza sativa*), overexpression of miR156 reduced the expression of *OsWRKY71*, resulting in enhanced expression of *MYB2* and *MYB3R-2*, thereby enhanced cold tolerance (Zhou and Tang, 2019). In Switchgrass (*Panicum virgatum*), overexpression of miR393 enhanced low temperature tolerance by regulating the auxin signaling pathway in switchgrass (Liu et al., 2017a). In tomato, Sha-miR319d enhances the cold tolerance by reducing the expression of *GAMYB-like1* and further changing ROS, heat and cold signal transduction (Shi et al., 2019). In citrus (*Citrus reticulata*), miR171 plays a key role in the adaptation to long-term B toxicity by regulating the expression of *SCARECROW* (Huang et al., 2019). In apples (*Malus pumila*), miR171i and its target gene *SCARECROW-LIKE PROTEINS26.1* improves drought stress tolerance by regulating antioxidant gene expression and ascorbic acid metabolism (Wang et al., 2020a). The miR171 regulates members of the *GRAS* gene family (Huang et al., 2017). GRAS proteins are widely taken part in signal transduction, growth and development, and stress in plants (Peng et al., 1997). In rice, *OsGRAS23* increases the transcription of genes related to stress response, especially the protein protection and antioxidant genes, and positively regulates the drought tolerance (Xu et al., 2015). In tomato, overexpression of *SlGRAS40* enhances drought resistance and salt resistance by regulating gibberellin (GAs) and auxin homeostasis (Liu et al., 2017b). The down-regulation of *SlGRAS10* improves the tolerance of abiotic stresses by regulating complex stress-induced gene expression in physiological modifications (Habib et al., 2021) and overexpression of *SlGRAS4* induces the expression of *SlCBF* and enhances the low temperature tolerance of tomato (Liu et al., 2020). *SlGRAS24* is the target gene of miR171d and involved in GAs signal transduction (Huang et al., 2017). Plant-specific GRAS protein family contains important regulatory parts of GA signaling that coordinately participate in plant growth and development (Shan et al., 2012). GRAS proteins have a subgroup that is a GA signaling repressor whose amino acid sequence in the N-terminal region is identical and is therefore called DELLA proteins (Chen et al., 2019). GAs induces the degradation of DELLA repressor protein and controls responses to cold stress (Lantzouni et al., 2020).

To further understand the function of miR171d in tomato, we obtained the up-regulated and down-regulated transgenic lines of Sly-miR171d by constructing the overexpression vector and the short tandem target mimic (STTM) vector. The results revealed the mechanism of Sly-miR171d in promoting cold resistance in tomato through GAs signal and provided help for cultivating stress tolerant germplasm.

## Materials and methods

### Plant expression vector construction and transformation

Silencing of mature Sly-miR171d was performed with STTM according to the method developed by Yan et al. (2012). The overexpression vector of Sly-miR171d was constructed according to the methods of Zhao et al. (2022a). After sequencing confirmation, all the correct constructs were transformed to Micro-Tom *via Agrobacterium*-mediated method. All primers and sequences are listed in Supplementary 1, 2.

### Sample treatment

Tomato (*Solanum lycopersicum* cv. Micro-Tom) plant materials used in this study were all homozygous T3 lines of Micro-Tom (MT). The plant growth chamber conditions were as follows: the ambient temperature was maintained at 23 ± 2°C; 16 h light/8 h dark alternate lighting; 60% humidity. Wild-type (WT) Micro-Tom tomato seeds were purchased with Nanjing Fengshuo Horticultural Co., Ltd. All fruit were selected with the same shape and size, without defect and disease. The fruit surface was soaked in 2% (V/V) sodium hypochlorite for 60 s to disinfect, washer with steam water, and dried. Green and red ripening tomato fruit (STTM-miR171d, WT, and miR171d-OE lines) with the same size, maturity and no mechanical damage were selected. The green ripening fruit were stored at 9°C for 30 days and randomly selected every 5 days. The shelf life was simulated at room temperature (25°C storage). Red ripening fruit were stored at 4°C for 25 days and randomly detected every 5 days. Each experiment was repeated two times.

### Chilling injury index

Chilling injury index was divided into five grades: 1 = no CI; 2 = slight damage (pitting covered <5% of fruit surface); 3 = moderate damage (pitting covers less than 25%, but >5% of the surface); 4 = severe damage (pitting coverage less than 50% but >25% of the surface); 5 = very severe damage (depression covers 50% of the surface). The CI was expressed as


$$\begin{array}{c} \text{CI}=\left[\sum \left(\text{CI}\text{scale}\right)\times \mathrm{fruit}\mathrm{number}\mathrm{at}\mathrm{that}\mathrm{scale}\right]/\\ \left(4\times \mathrm{total}\,\mathrm{number}\,\mathrm{of}\,\mathrm{fruit}\,\mathrm{in}\,\mathrm{the}\,\mathrm{group}\right). \end{array}$$

Each group selected 12 tomato fruit to evaluate the degree of CI.

### Electrolyte leakage, firmness, and total soluble solids of tomato fruit

Electrolyte leakage (EL) was detected according to the method of Li et al. (2016). FE30 conductivity meter (Mettler Toledo Instrument Co., Ltd., China) was used to measure the EL of fruit. Select 10 circular pulp tissues (10 mm straight × 4 mm thick in diameter) from the equatorial region of five tomato fruit and measure P_1_ by shaking in 30 ml of distilled water for 20 min. P_2_ is then measured after the sample has been boiled for 15 min. where P_1_ is the initial conductivity value and P_2_ is the final conductivity value.

Fruit firmness was detected according to the method of El-Mogy et al. (2018). Measuring fruit firmness with a texture analyzer (TA; XT Plus, Stable Micro Systems, United Kingdom) by a 2 mm flat probe at 3 points in the equatorial region, with hardness values of three fruit repeated at a time.

Total soluble solids (TSS) was detected according to the method of Shan et al. (2022). TSS in tomato pulp was determined at room temperature using a hand-held refractometer (WY032R; Chengdu Optical Instrument Factory, China). The results are expressed as %.

### Determination of GA_3_ content

The tomato samples were ground to homogenate and determined according to plant GA_3_ ELISA kit (Jiangsu Meibiao Biotechnology Co., Ltd., China).

### RNA isolation and quantitative real-time PCR analysis

The kits (Tiangen, Beijing, China) were used for total RNA extraction, first-strand cDNA synthesis and real-time quantitative PCR. The expression of Sly-miR171d, *SlGRAS24*, *COR, CBF1*, *GA20ox1, GA3ox1, GA2ox1*, and *GAI* were calculated by the 2^–ΔΔCt^ method. The internal reference gene is *Actin.* The qRT-PCR primers are supplemented in Supplementary Table 1. Two grams of fruit pulp tissue were taken from three tomato fruit, ground in liquid nitrogen, and RNA was extracted according to the kit.

### Statistical analysis

Values were expressed as means ± standard deviation (SD). Data analysis using SPSS 20.0 (SPSS, Chicago, IL, United States). A one-way analysis of variance (ANOVA) was performed to test significant differences among the means (*P* < 0.05).

## Results

### Characterization of STTM-miR171d and miR171d-OE fruit

Stable transformation of tomato plant STTM-miR171d and miR171d-OE lines were produced by *Agrobacterium*-mediated method. A total of 25 independent tomato transgenic lines (16 STTM-miR171d and 9 miR171d-OE plants) were detected in the T1 generation. After PCR amplification with *hygromycin* (STTM-miR171d lines) and *kanamycin* (miR171d-OE lines), cell lines with target bands were detected by agarose gel electrophoresis (Supplementary 1). This indicated that the vector had been successfully integrated into the tomato chromosome. Expression analysis of miR171d-OE and STTM-miR171d in T3 transgenic plants and identify them as transgenic fruit, and selected as experimental materials. Green tomato fruit was harvested at 35 dpa and red tomato fruit was harvested at 45 dpa.

### Chilling injury index

**FIGURE 1:**
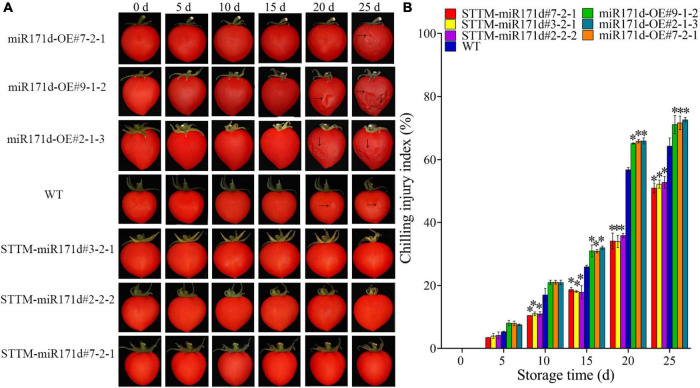
Surface CI symptoms and CI index of red ripe tomato fruit. **(A)** Symptoms of CI on the surface of tomato fruit. **(B)** CI index. The error bar represents the independent repeat SDs (*n* = 3). The asterisk indicates that the data difference with *P* < 0.05 is statistically significant.

**FIGURE 2:**
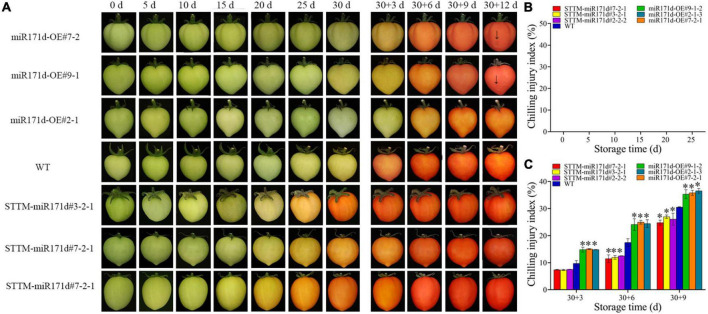
Surface CI symptoms and CI index of green ripe tomato fruit. **(A)** Symptoms of CI on the surface of tomato fruit. **(B,C)** CI index. The error bar represents the independent repeat SDs (*n* = 3). The asterisk indicates that the data difference with *P* < 0.05 is statistically significant.

Silencing of Sly-miR171d with STTM delayed CI symptoms in fruit compared to WT (Figures 1A, 2A). The red miR171d-OE tomato showed obvious epidermal atrophy on the 20 days under the condition of 4°C. With the storage time Prolonged, the phenomenon of skin atrophy deepened. WT tomato fruit showed CI symptoms after 20 days, and the symptoms worsened at 25 days, while STTM-miR171d fruit did not show obvious abnormalities on the surface of fruit after 25 days of storage. During the green ripening stage, the miR171d-OE tomato fruit were stored at 9°C for 30 days, and the skin wrinkles appeared after the 12 days of the shelf life. WT tomato fruit and STTM-miR171d fruit did not show obvious symptoms of CI.

In the red ripening stage, at 4°C for 25 days, the CI of miR171d-OE, STTM-miR171d, and WT tomato fruit were 71.80, 52.02, and 64.30%. The CI of WT tomato fruit was 12.28% higher than that of STTM-miR171d (Figure 1B). The CI of miR171d-OE, STTM-miR171d, and WT tomato fruit were 35.89, 25.96, and 30.53%, respectively, when green tomato fruit were refrigerated and stored at 25°C for 9 days. The CI of WT tomato fruit was 4.57% higher than that of STTM-miR171d (Figure 2C).

### Electrolyte leakage, firmness, total soluble solids, and GA_3_ content of tomato fruit

**FIGURE 3:**
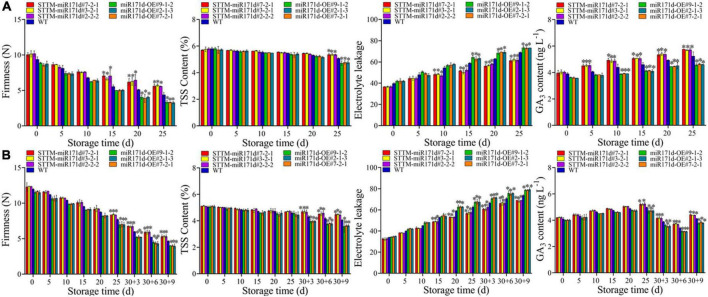
Electrolyte leakage, Firmness, TSS, and GA_3_ content of tomato fruit in red ripening stage **(A)** and green ripening stage **(B)**. The error bar represents the independent repeat SDs (*n* = 3). The asterisk indicates that the data difference with *P* < 0.05 is statistically significant.

The accumulation of EL in red and green fruit increased under low temperature stress. At 4°C for 25 days, The EL of miR171d-OE tomato red fruit was 4.18 higher than WT, and that of STTM-miR171d tomato was 7.09 lower than WT. After the green tomato fruit was refrigerated and stored at 9°C, the EL content of STTM-miR171d fruit was 4.83 lower than WT. The EL content of miR171d-OE fruit was 5.23 higher than WT (Figure 3).

In tomato fruit, firmness decreased gradually with storage time. After 25 days of low temperature stress, the firmness of red STTM-miR171d tomato fruit decreased to 5.65 N. The hardness of miR171d-OE decreased to 3.32 N and that of WT was 4.38 N. The firmness of STTM-miR171d fruit was 1.29 times that of WT. After storage at 25°C for 9 days at green ripening stage, fruit hardness of STTM-miR171d, miR171d-OE, and WT were 5.32, 3.99, and 4.66 N (Figure 3).

The TSS in tomato fruit gradually decreased. The red fruit is stored for 25 days, miR171d-OE fruit decreased from 5.75 to 4.74%, and WT fruit decreased from 5.81 to 5.07%, while STTM-miR171d fruit still maintained 5.36% at 25 days. The TSS content of green tomato fruit showed significant differences after the shelf life, miR171d-OE fruit decreased from 5.08 to 3.61%, WT fruit decreased from 5.05 to 4.06%, while STTM-miR171d fruit remained at 4.48% at the end (Figure 3).

During low temperatures, the GA_3_ content of red tomato fruit showed an overall upward trend, but the content of miR171d-OE was lower than that of STTM-miR171d. After 25 days of storage, the contents of STTM-miR171d, WT, and miR171d-OE increased to 5.73, 5.24, and 4.61 ng L^–1^. The GA_3_ content of green tomato fruit showed an increasing trend throughout the storage period and gradually decreased during the shelf life. During the shelf life, the fruit content of STTM-miR171d decreased from 5.23 to 4.40 ng L^–1^, and the fruit content of WT decreased from 4.97 to 4.13 ng L^–1^. The fruit content of miR171d-OE decreased from 4.73 to 3.81 ng L^–1^, and the content of STTM-miR171d was 6.54% higher than WT (Figure 3).

### The expressions of *CBF1* and *COR* in tomato

**FIGURE 4:**
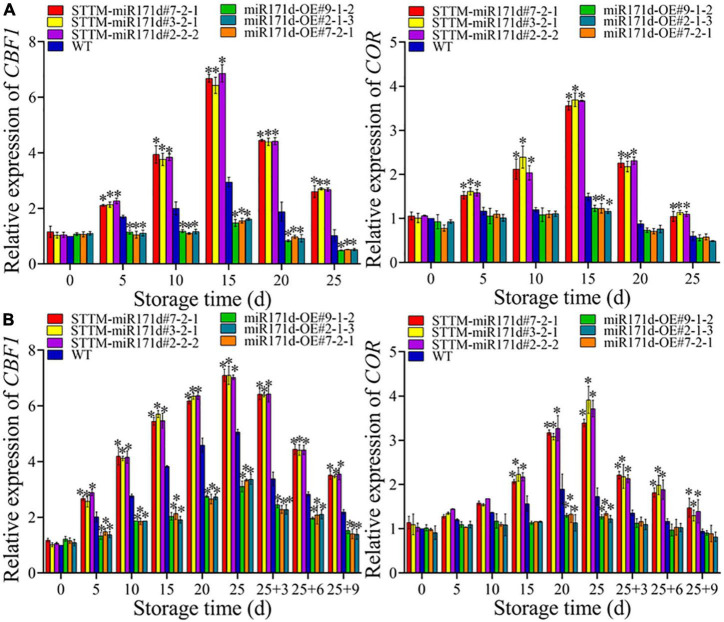
The expression levels of *CBF1* and *COR* genes in red ripe tomato fruit **(A)** and green ripe tomato fruit **(B)**. The error bar represents the independent repeat SDs (*n* = 3). The asterisk indicates that the data difference with *P* < 0.05 is statistically significant.

In red tomato fruit, the expression of *COR* genes and *CBF1* genes showed an upward trend from 0 to 15 days and then sharply decreased, as shown in Figure 4A. At 25 days, the *CBF1* gene expression levels of STTM-miR171d#7-2-1, STTM-miR171d#3-2-1, and STTM-miR171d#2-2-2 were 2.55, 2.65, and 2.62 times than WT. The *COR* gene expression of STTM-miR171d#7-2-1, STTM-miR171d#3-2-1, and STTM-miR171d#2-2-2 were 1.75, 1.90, and 1.83 times than WT, respectively. *CBF1* and *COR* genes in green tomato fruit showed an increasing trend during storage, but sharply decreased during shelf life, as shown in Figure 4B. At the end of 9 days shelf life, the *CBF1* gene expression levels of STTM-miR171d#7-2-1, STTM-miR171d#3-2-1, and STTM-miR171d#2-2-2 were 1.61, 1.59, and 1.63 times than WT. The *COR* gene expression of STTM-miR171d#7-2-1, STTM-miR171d#3-2-1, and STTM-miR171d#2-2-2 were 1.56, 1.38, and 1.48 times than WT (Figure 4).

### The expressions of miR171d and *SlGRAS24* in tomato

**FIGURE 5:**
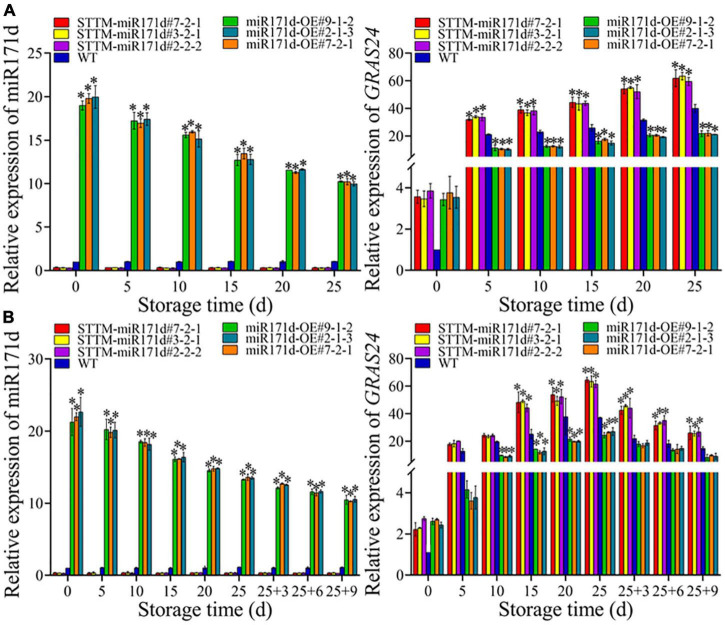
The expression levels of Sly-miR171d and *SlGRAS24* genes in red ripe tomato fruit **(A)** and green ripe tomato fruit **(B)**. The error bar represents the independent repeat SDs (*n* = 3). The asterisk indicates that the data difference with *P* < 0.05 is statistically significant.

During the low temperature, the expression level of *miR171d* in STTM-miR171d fruit was not significant and lower than that in WT fruit. Red tomato fruit was refrigerated at 4°C, STTM-miR171d#7-2-1, STTM-miR171d#3-2-1, and STTM-miR171d#2-2-2 the expression of miR171d were 0.34, 0.30, and 0.34. After green tomato fruit was refrigerated at 9°C and stored at 25°C for 9 days, the expression levels of miR171d in STTM-miR171d#7-2-1, STTM-miR171d#3-2-1, and STTM-miR171d#2-2-2 were 0.35, 0.36, and 0.31. The miR171d gene was significantly expressed in tomato fruit miR171d-OE, and the gene expression decreased with an extended storage period. At 25 days in red tomato fruit, the miR171d of miR171d-OE#9-1-2, miR171d-OE#7-2-1, and miR171d-OE#2-1-3 were 9.76, 9.74, and 9.50 times that of WT. After the shelf life of green tomato fruit, the miR171d in miR171d-OE#9-1-2, miR171d-OE#7-2-1, and miR171d-OE#2-1-3 were 9.52, 9.34, and 9.59 times than WT (Figure 5).

In red STTM-miR171d tomato fruit the expression of *GRAS24* gene increased with storage time under low temperature stress. Red tomato fruit after 25 days, the expression of *GRAS24* in STTM-miR171d#7-2-1, STTM-miR171d#3-2-1, STTM-miR171d#2-2-2, and WT were 61.89, 63.38, 59.49, and 40.06. The expression levels of *GRAS24* in red fruit of miR171d-OE#9-1-2, miR171d-OE#7-2-1, and miR171d-OE#2-1-3 were 21.78, 22.07, and 21.15. Under low temperature stress in green STTM-miR171d tomato, the expression of *GRAS24* gene increased with the storage time during the storage period, and the shelf life decreased gradually. At the end of the shelf life at 25°C, the expression of *GRAS24* in STTM-miR171d#7-2-1, STTM-miR171d#3-2-1, STTM-miR171d#2-2-2, and WT were 26.06, 25.50, 26.65, and 14.78. The expression levels of *GRAS24* in green fruit of miR171d-OE#9-1-2, miR171d-OE#7-2-1, and miR171d-OE#2-1-3 were 8.20, 9.76, and 9.07 (Figure 5).

### The expressions of *GA20ox1*, *GA3ox1*, *GA2ox1*, and *GAI* in tomato

**FIGURE 6:**
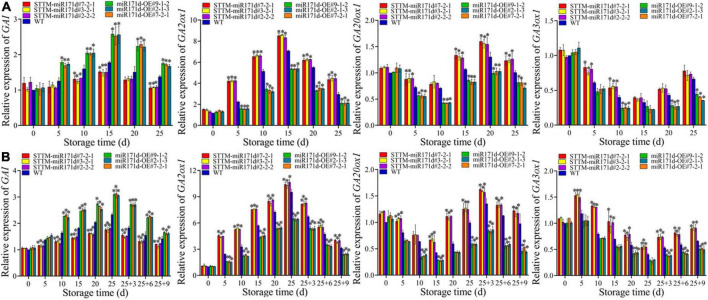
The expression levels of *GA20ox1*, *GA3ox1*, *GA2ox1*, and *GAI* genes in red ripe tomato fruit **(A)** and green ripe tomato fruit **(B)**. The error bar represents the independent repeat SDs (*n* = 3). The asterisk indicates that the data difference with *P* < 0.05 is statistically significant.

*GA20ox1*, *GA3ox1*, and *GA2ox1* are key genes that affect bioactive GA levels in plants. *GA20ox1* and *GA3ox1* are synthetic genes, and *GA2ox1* is the catabolic gene. At 25 days in red tomato fruit, the expression of *GA20ox1* and *GA3ox1* genes in STTM-miR171d were 1.24 and 1.12 times that of WT. After 9 days of shelf life, the expression of *GA20ox1* and *GA3ox1* genes in STTM-miR171d were 1.23 and 1.38 times that of WT. The expression of the *GA2ox1* gene increased rapidly and then decreased during the storage period. At 25 days of red tomato fruit, the *GA2ox1* gene expression level in STTM-miR171d fruit was 1.50 times that of WT. Green tomato after 9 days of shelf life, the expression level of *GA2ox1* gene in STTM-miR171d fruit was 1.25 times that of WT (Figure 6).

In tomato fruit the expression of the *GAI* gene first increased and then decreased. The expression of *GAI* in red tomato fruit reached the peak at 15 days. On 25 days, miR171d-OE #9-1-2, miR171d-OE #7-2-1, and miR171d-OE #2-1-3 were 1.26, 1.25, and 1.21 times than those in WT fruit, respectively. The expression of *GAI* in green tomato fruit reached its peak at the end of storage period. After 9 days of shelf life, miR171d-OE #9-1-2, miR171d-OE #7-2-1, and miR171d-OE #2-1-3 were 1.18, 1.14, and 1.14 times than WT, respectively (Figure 6).

## Discussion

Refrigeration is currently the most commonly used technique for extending the shelf life of postharvest fruit. However, most tropical and subtropical fruit are susceptible to CI during refrigeration (Wang et al., 2021). As important regulatory factors of various physiological processes in plants, miRNA also plays a significant role in plants resistance to cold tolerance (Zhou and Tang, 2019). Low temperature stress can increase membrane permeability and damage the membrane system, leading to cell metabolism and dysfunction in plants (Mekontso et al., 2021; Wang et al., 2022). EL is one of the main outcomes caused by CI, and elevated EL is considered an important marker of cell membrane damage, with lower EL indicating higher membrane integrity (Duan et al., 2022). CI index reflects the degree of CI symptoms and judges cold tolerance, and the development of CI symptoms increases with long-term storage (Zhang et al., 2022). The contents of TSS reflects the intracellular mass and solute concentration, and is a protective substance in the cells under the conditions of CI, and its content is positively correlated with the cold resistance of most plants (Jia et al., 2018). The present results showed that STTM-miR171d tomato fruit surface did not change significantly, WT fruit showed epidermis shrank, while miR171d-OE tomato fruit showed epidermis atrophy and surface depression. The accumulation of EL in tomato fruit increased with storage time, and the accumulation of EL in STTM-miR171d fruit did not increase significantly. TSS content in STTM-miR171d fruit was higher than that of miR171d-OE and WT fruit, and remained at a higher level. Inhibition of Sly-miR171d can slow down the development of CI symptoms in tomato fruit, reduce the accumulation of EL, maintain high cell membrane integrity and high TSS content to delay the decline of fruit tissue firmness. Therefore, silencing Sly-miR171d improved the cold resistance of tomato fruit.

**FIGURE 7:**
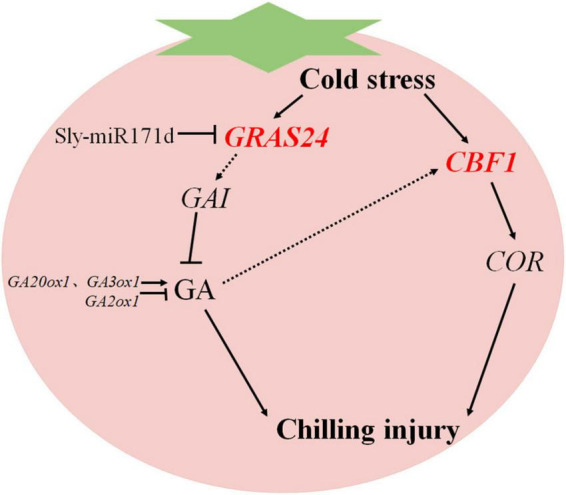
A simplified model of the regulatory mechanism of Sly-miR171d on cold tolerance of tomato fruit stored at low temperature.

GA_3_ is a natural plant hormone that plays a crucial role in various abiotic stresses (Alonso-Ramírez et al., 2009). GA inactivation and positive and negative feedback regulation of GA biosynthesis to maintain GA homeostasis (Hedden and Thomas, 2012). The down-regulation of GA biosynthesis genes *GA20ox1* and *GA3ox1* in tomato fruit stored at low temperature is related to the decrease of GA content (Zhu et al., 2016). Exogenous SA treatment of tomato fruit induced *GA3ox1* expression, increased GA_3_ content, stimulated the degradation of DELLA protein, and alleviated CI symptoms in tomato fruit (Ding et al., 2015). GRAS transcription factors are essential in plant responses to stress. DELLA belongs to the GRAS protein family, and GA can stimulate DELLA accumulation, inhibit protein degradation, and control key developmental processes to enhance stress tolerance (Achard et al., 2008). Exogenous GA_3_ may resist phytoplasma infection of potato purple top disease by down-regulating the *DELLA* gene (Ding et al., 2013). Under cold stress, *SlPIF4* activates the *SlDELLA* gene and inhibits GA biosynthesis to partially enhance the cold tolerance of tomato plants (Wang et al., 2020b). Overexpression of tomato transcription factor *SlGRAS40* affects auxin and GA pathways in the process of tomato nutrition and reproduction, promotes the accumulation of DELLA protein, and enhances plants stress resistance (Liu et al., 2017b). In the study, the expression levels of key GA metabolizing genes and signal transduction genes in red and green ripe tomato fruit were analyzed. After low temperature storage, the expression level of target gene *GRAS24* was positively correlated with GA biosynthesis key genes *GA20ox1*, *GA3ox1*, and *GA2ox1*. It was negatively correlated with the DELLA protein gene *GAI*. These results suggested that Sly-miR171d negatively regulated target gene *GRAS24*, increased GA content in tomato fruit and down-regulated DELLA protein gene *GAI*. Conversely, the expression of key genes *GA20ox1* and *GA3ox1* for GA biosynthesis and the GA decomposition gene *GA2ox1* were up-regulated. The target gene *GRAS24* may inhibit growth by downregulating the relative expression of *GAI* or degrading DELLA protein, thereby activating the cold tolerance of tomato fruit (Figure 7).

The CBF pathway is essential in plant resistance to cold stress. The transcript level of *CBF1* is positively correlated with tomato chilling resistance and negatively correlated with tomato CI index, which is for assessing the chilling resistance of tomato (Zhao et al., 2011). The expression of CBF-related genes in *Arabidopsis thaliana* can actively regulate low temperature stress resistance (Jiang et al., 2020). Overexpression of *CmICE2* in *Arabidopsis* induced up-regulation of *CBF* and *COR* genes, and enhanced the response to freezing stress (Zhang et al., 2020). Overexpression of *AtCBF1* in potato induces physiological modifications associated with cold adaptation to enhance freezing resistance (Pino et al., 2008). CBF has been shown to fully activate *COR* gene expression and induce resistance to low temperature stress (Stockinger et al., 1997). Application of exogenous GA_3_ increased the expression of the *CBF1* gene and improved low temperature tolerance of tomato fruit by regulating GA catabolism through the CBF pathway (Zhu et al., 2016). Exogenous SA induced the expression of *CBF1*, which in turn induced the expression of the *GA2ox1* gene, thereby activated the biosynthesis of GA and alleviated the CI of tomato fruit during low temperature storage (Ding et al., 2016). In this study, *CBF1* and *COR* were higher expressed in STTM-miR171d than that in miR171-OE and WT fruit, which was consistent with their phenotype. The expression patterns of *CBF1* and *GA2ox1* are similar, and the *CBF* pathway may enhance the low temperature tolerance of tomato fruit by regulating GA catabolism (Figure 7).

## Conclusion

In conclusion, silencing of Sly-miR171d and enhancing the expression of target gene *GRAS24* effectively maintained the stability of cell membrane and increased the GA_3_ level in tomato fruit under low temperature, thereby improved postharvest chilling tolerance of tomato fruit.

## Data availability statement

The datasets presented in this study can be found in online repositories. The names of the repository/repositories and accession number(s) can be found in the article/Supplementary material.

## Author contributions

ZX, XX, and SL conceived and designed the experiments. ZX, TH, and KZ conducted the experiments. ZX, TH, KZ, LM, HS, ZZ, XX, and SL wrote and edited the manuscript. All authors approved the publication of final version of the manuscript.
