# Supplementary material for: Silencing of Sly-miR171d increased the expression of GRAS24 and enhanced postharvest chilling tolerance of tomato fruit
Source: Front Plant Sci. 2022 Sep 9;13:1006940. doi: 10.3389/fpls.2022.1006940 (PMC9500411; doi:10.3389/fpls.2022.1006940)
Supplement: Supplementary file 1 [file Data_Sheet_1.ZIP › Supplementary 1.pdf]

Supporting Information

Gene	Primer	Sequence (5' -3')
GAI	Forward Primer	TGATGCGACTATACTTGATATAAG
	Reverse Primer	GGGTTAATCTGTTTAATAGAGTTC
SlGA20ox1	Forward Primer	CTCATTTCTAATGCTCATCGT
	Reverse Primer	TGCAGATGATTCTTTCTTAGCG
SlGA3ox1	Forward Primer	GGCATTAGTAGTTAATATAGGTGA
	Reverse Primer	AAATAAGCTACAGAAAGTCGATA
SlGA2ox1	Forward Primer	GGCATGTAAGATATTAGAATTGA
	Reverse Primer	TTAATCCGTAGTAGAGAATCAGA
TOM-U6	Forward Primer	TCTAACAGTGTAGTTTGTCCCTTCG
	Reverse Primer	TTGTGCGTGTCATCCTTGC
RT-miR171d	Primer	GTCGTATCCAGTGCAGGGTCCGAGGTATTCG CACTGGATACGACGTGATA
SlGRAS24	Forward Primer	GCTTGAGGTCTTGGATGG
	Reverse Primer	TGTGGATGGTGAGGCTAA
CBF1	Forward Primer	GGCAGAAGGACTTATGCTACC
	Reverse Primer	ACCCAACAAGTTTCTGTTCATGT
COR	Forward Primer	GGCATCCGTTGAAGAGACTG
	Reverse Primer	GGCATCCGTTGAAGAGACTG
Actin	Forward Primer	TGTGTTGGACTCTGGTGATGGTGT
	Reverse Primer	ATCCAAACGAAGAATGGCATGCGG
Sly-miR171d	Forward Primer	CGGCGGTTGAGCCGCGCCAA
	Reverse Primer	GTGCAGGGTCCGAGGTATTC

Table.S1 All primers sequences The letters F and R indicate forward (F) and reverse (R) primers of the strand gene sequence.

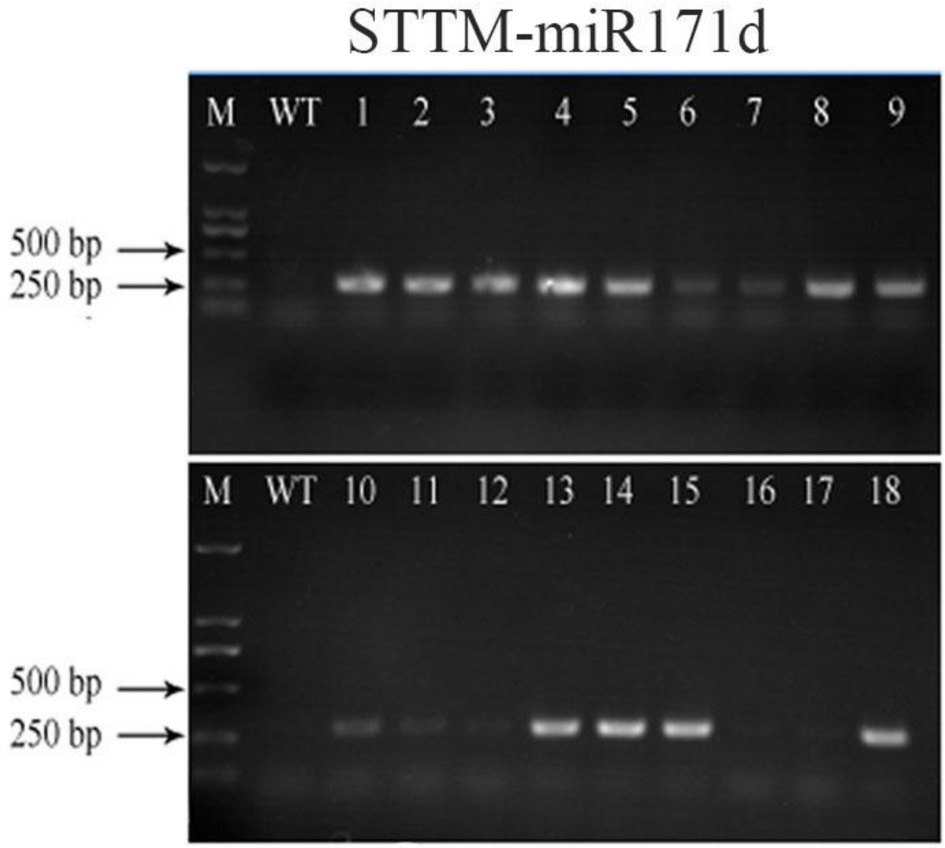

Fig.S1 The PCR identification of STTM-miR171d transgenic plants. WT: wild-type plant; 1-18: positive transgenic plant.

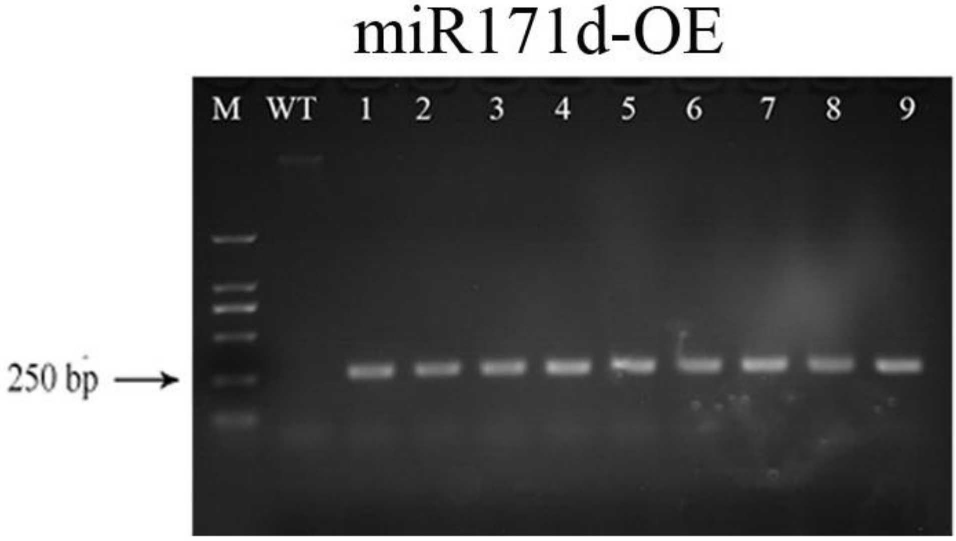

Fig.S2 The PCR identification of miR171d-OE transgenic plants. WT: wild-type plants; 1-9: positive transgenic plants.
