# Supplementary material for: Silencing of Sly-miR171d increased the expression of GRAS24 and enhanced postharvest chilling tolerance of tomato fruit
Source: Front Plant Sci. 2022 Sep 9;13:1006940. doi: 10.3389/fpls.2022.1006940 (PMC9500411; doi:10.3389/fpls.2022.1006940)
Supplement: Supplementary file 1 [file Data_Sheet_1.ZIP › Supplementary 2.pdf]

## Supporting Information

The STTM-miR171d sequence is represented in red font, and the 48 nt sequence is represented by a separate yellow shadow. The BsaI and Eco31I restriction sites are shown in bold green. The green shadows represent 2 ×35PS and T35PS respectively.

> STTM-miR171d

CTTCAAAGCAAGTGGATTGATGTGATATCTCCACTGACGTAAGGGATGACG
CACAATCCCACTATCCTTCGCAAGACCCTTCCTCTATATAAGGAAGTTCATT
TCATTTGGAGAGAACACGGGGGACTTTGA**GGCAAC**GTGATATTGGCTACG
CGGCTAAGTTGTTGTTGTTATGGTCTAATTTAAATATGGTCTAAAGAAGAAG
AATGTGATATTGGCTACGCGGCTCAA**TGTAAC**TAGCTCTGTCTTCAGTACTG
GGCCCGAAGACTGACCAGCTCGAATTCCCCGATCGTCAAACATTGCAATA
AGTA

The sequences of pri-miR17d. Green shades indicate the precursor sequence of miR171d. Yellow shades and bold indicate the miR171d stem-loop sequence and the mature miR171d sequences.

>pri-miR171d

CCACCTAGAATATTCTTCTGGAAAAAAAATTCTATCCGTTCTATCATAATC
CGCGATATATTAGATCGTTCTCACTCACCGCTATGTCAATATCTTTTTTAA
AGTTTGAATGGTTGATCAAAATATTTTGGGATTAAAATGGAAGAAGCGAT
GTTGGTGAGGTTCAATCCGAAGACGAATTTATGCTTATTTTCGTAAAGAAC
GATCTCAGA**TTGAGCCGCGCCAATATCAC**TTCTTATTTTCATTCCGTATT
TCCCATCTGATGACCAATTAATTCCAAAATAGCGCATTATGAAATTCATTT
CTTTAAAAGGCAACGTTCTCAATAAAATATATTTCTTTCTCAATGGCTCAA
ACCCGATATCACTTCCTATTATGTATCATTTCATTCGTTCATTTTTCTTGTC
CATGGTAAGTTTACATTTGTCTACAAGGCTTCTCCTT
